# Cryopreventive temperatures prior to chemotherapy

**DOI:** 10.1007/s12032-023-01989-9

**Published:** 2023-04-14

**Authors:** A. Ibrahim, E. Camci, L. Khairallah, M. Jontell, J. Walladbegi

**Affiliations:** 1 Department of Oral and Maxillofacial Surgery, Institute of Odontology, The Sahlgrenska Academy, University of Gothenburg, PO Box 450, 405 30 Gothenburg, Sweden; 2 Department of Oral Medicine and Pathology, Institute of Odontology, The Sahlgrenska Academy, University of Gothenburg, Gothenburg, Sweden

**Keywords:** Cryotherapy, Intraoral cooling device, Oral mucositis, Tolerability

## Abstract

The superiority of oral cryotherapy (OC) for prevention of chemotherapy-induced oral mucositis (OM) has been demonstrated in several trials. In clinical settings, cooling is usually initiated prior to the chemotherapy infusion. It then continues during the infusion, and for a period after the infusion has been completed. While the cooling period post-infusion depends on the half-life of the chemotherapeutic drug, there is no consensus on when cooling should be initiated prior to the infusion. The lowest achieved temperature in the oral mucosa is believed to provide the best condition for OM prevention. Given this, it was of interest to investigate when along the course of intraoral cooling this temperature is achieved. In total, 20 healthy volunteers participated in this randomized crossover trial. Each subject attended three separate cooling sessions of 30 min each, with ice chips (IC) and the intraoral cooling device (ICD) set to 8 and 15 °C, respectively. At baseline and following 5, 10, 15, 20 and 30 min of cooling, intraoral temperatures were registered using a thermographic camera. The greatest drop in intraoral temperature was seen after 5 min of cooling with IC, ICD^8°C^ and ICD^15°C^, respectively. A statistically significant difference, corresponding to 1.4 °C, was seen between IC and the ICD^15°C^ (*p* < 0.05). The intraoral temperature further declined throughout the 30 min of cooling, showing an additional temperature reduction of 3.1, 2.2, and 1.7 °C for IC, ICD^8°C^ and ICD^15°C^, respectively.

## Introduction

Oral mucositis (OM) has been reported as one of the worst adverse effects associated with cancer therapy [1], affecting up to 80% of all patients receiving high-dose chemotherapy in conjunction with haemopoietic stem-cell transplantation [2]. The risk of developing OM is further increased among patients with head and neck tumors where radiation is used as single therapy or in combination with surgery to eradicate the cancerous tissue. In this cohort nearly all patients have clinically manifested OM [3].

Chemotherapy-induced OM usually affects the non-keratinized mucosae and is in its mildest form seen as erythematous regions with an intact epithelial lining [4]. In contrast, severe OM is characterized by ulcerations with exposed underlying connective tissue [5, 6]. This painful condition may last for weeks or even months if secondary infected [7]; and it can represent a portal of entry for systemic infections that can lead to sepsis and death. OM is further associated with undernourishment, weight loss, the use of feeding tubes or total parenteral, and an increased need for intravenously administered opioids for pain relief [8]. Taken together, these symptoms along with their related sequelae can lead to impaired quality of life, and may negatively affect the outcome of the medical treatment [1, 9–12].

Several methods have been proposed for prevention of chemotherapy-induced OM [13–15]. Nevertheless, oral cryotherapy (OC) using ice chips (IC) continues to be the best alternative [16]. Unfortunately, the use of IC is associated with considerable discomfort such as headache, nausea, and chills [17]. In addition, the use of IC requires that it is made from water of good quality, so that there is no risk of contamination by microorganisms, with the consequent risk of infections in already immunosuppressed patients. To overcome the limitations of IC, an intraoral cooling device (ICD) has been developed [18]. The ICD is made up of a closed conduit system with continuously circulating water; and can be set to operate at different temperatures. In a randomized controlled trial, the ICD set to 8 °C was compared to IC, confirming the efficacy of OC for prevention of OM [19]. However, the ICD was better tolerated and showed superiority to IC in the subgroup with lymphoma patients.

Intraoral cooling is initiated prior to the chemotherapy infusion. The cooling procedure then continues during the infusion, and for a period after the infusion has been completed. While the cooling period post-infusion depends on the half-life of the chemotherapeutic drug [20], there is no consensus on when cooling should be initiated prior to the infusion. Clinical protocols may vary and anything between minutes to hours has been reported, usually without any evidence-based justification [21–24].

The lowest achieved temperature in the oral mucosa is believed to provide the best conditions to prevent OM. Given this, it was of interest to investigate when along the course of intraoral cooling this temperature was achieved, and if there was any difference between different cooling modalities and temperatures.

Thus, the aim of the present study was to study intraoral temperatures during a clinically relevant time of cooling, using IC and the ICD set to 8 or 15 °C.

## Subjects and methods

### Subjects

All subjects were healthy dental students and dentists recruited from the Sahlgrenska Academy, University of Gothenburg, Gothenburg, Sweden. To participate in this study, the following inclusion criteria had to be fulfilled: (i) willing and able to provide informed written consent, (ii) age ≥ 18 years, (iii) no medical diagnosis established by a physician, and (iv) no use of any medication affecting the cardiovascular system. Exclusion criteria were: (i) use of tobacco (e.g., cigarettes, e-cigarettes, or Swedish snuff); (ii) presence of oral mucosal lesions, (iii) previous participation in studies evaluating cryotherapy, and (iv) basic hemodynamics deviating from the normal values.

### Study design

This randomized crossover trial was conducted between August and September 2021. All subjects were informed to attend three separate intraoral cooling sessions. The order in which the cooling sessions were carried out were randomized for each subject, using a free online randomization tool (https://www.randomizer.org/). The intraoral cooling sessions continued for 30 min per session with either ice chips (IC) or the intraoral cooling device (ICD) set to 8 or 15 °C. There was a washout period of at least 24 h between each cooling session to assure that baseline intraoral temperatures were recovered. All cooling sessions were carried out at the Institute of Odontology, The Sahlgrenska Academy, University of Gothenburg, Gothenburg, Sweden.

### Instruments

**Fig. 1:**
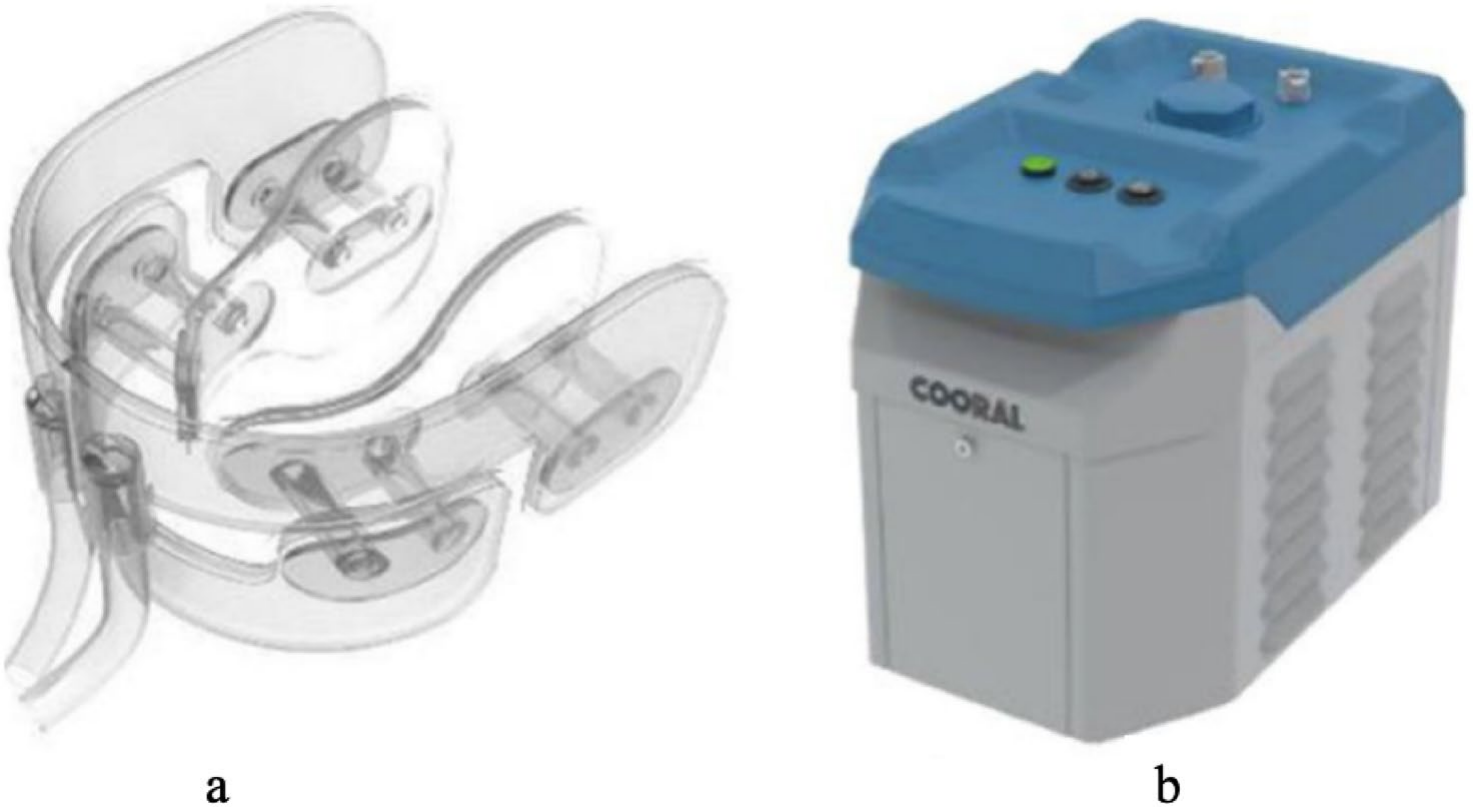
The Cooral® System. **a** schematic illustration of the intraoral cooling device; **b** the portable thermostat unit. Reprinted and modified with permission from Walladbegi J., Gellerstedt M., Svanberg A., Jontell M. Innovative intraoral cooling device better tolerated and equally effective as ice cooling. *Cancer Chemother Pharmacol*. 2017 Nov; 80(5):965–72. (http://creativecommons.org/licenses/by/4.0/)

IC were produced using a commercial ice maker (Porkka KF145 Flake Ice Machine, Oulu, Finland), and stored in a styrofoam box during the cooling sessions. The IC temperature was approximately −0.5 °C upon exposure. The ICD (Cooral® Mouth device; Fig. 1a) was provided by BrainCool AB, Lund, Sweden. The design of the ICD allows cooling of the buccal mucosae, lips, floor of the mouth, tongue, gingivae, and hard palate. The ICD is a single-use device and was available in one size (large). It consists of a closed conduit system with continuously circulating water delivered by a portable thermostat unit (Cooral® System; Fig. 1b). The thermostat unit delivers water with a flowrate of 0.25 l/min (±0.1 l/min) at a pre-set temperature between 6 and 22 °C (±2 °C). A thermographic camera, FLIR E60 (bx) (FLIR Systems Inc., Wilsonville, OR, USA) with the following specifications were used to capture intraoral images; IR sensor with 4.800 measurement pixels, object temperature range of −10 °C to +150 °C, measurement accuracy of ±2 °C, thermal sensitivity of < 0.10 °C, spectral range of 7.5–14 μm, and minimal focus distance of 0.15 m [25]. The thermal images were downloaded, computer stored as JPEG files, and analyzed using the associated FLIR tools software (version 6.4). The temperatures were reflected with colors in each image, ranging from black for the coldest areas, to white for the highest temperatures with a temperature scale next to each image corresponding to the colors. Using the software function, areas of interest were delineated to measure the mean intraoral temperature for each location.

### Procedures and data collection

All measurements were performed in the same examination office (ambient temperature 22 ±2 °C). Following inclusion, medical history was gathered, body mass index (BMI) [kg/m^2^] was calculated, and an intraoral examination was carried out by the investigators to confirm a healthy oral mucosa with no ulcerations or other pathological conditions that could affect the cooling procedures. Basic systemic hemodynamics, including heart rate, systolic-, and diastolic blood pressure were measured at baseline, in the left upper arm with the subject in a sitting position, using a digital blood pressure monitor (Omron, HigashiNoda, Osaka, Japan). Subjects were informed not to consume any food or drink up to 30 min prior to the cooling sessions.

At baseline and following 5, 10, 15, 20 and 30 min of cooling with each of the three cooling sessions, intraoral thermographic images were captured in the following eight intraoral mucosal locations; right buccal, left buccal, upper labial, lower labial, dorsal tongue, ventral tongue, hard palate, and floor of the mouth. Two dental mirrors made of stainless steel were used during the imaging process to access the surfaces of interest.

For the cooling sessions using IC, subjects were informed to insert an ounce of ice and move the IC around in the mouth throughout the cooling session, except for when the thermal images were captured. They were also briefed to rinse the melted ice slurry that was obtained before it was swallowed or expectorated. Prior to the cooling sessions with the ICD, all subjects were supervised in how to insert and use the ICD.

All procedures were standardized throughout the study and the images were analyzed by a dentist blinded to the cooling procedures.

### Statistical analyses

Normality assumption was controlled using the Shapiro-Wilks and a Gaussian distribution was confirmed for all the tested variables. Descriptive data were presented with mean (x̄) and standard deviation (SD). To determine any statistically significant differences in temperature reduction after 5 min of cooling between IC, and ICD^8°C^ or ICD^15°C^, One-way analysis of variances (ANOVA) was performed, followed by a post hoc test, Tukey, for multiple comparisons. A two-sided paired samples Student’s t-test was performed to assess any statistical differences between 5 and 30 min of cooling within each cooling method. A *p* value ≤ 0.05 was considered statistically significant. The calculations were performed using the IBM® SPSS® Statistics software package (IBM SPSS Statistics version 24, IBM, Armonk, NY).

## Results

In total, 20 out of 25 (80%) fulfilled the inclusion criteria. The remaining five subjects (20%) were excluded due to the use of Swedish snuff or medication that could possibly affect the outcome of the study. Subject characteristics, including basic hemodynamics and procedural times are summarized in Table 1. All subjects endured 30 min of cooling with all three cooling procedures, accounting for a total of 60 cooling sessions during the study. In total, 2880 thermographic images were captured, of which 13 were excluded from the final analysis due to poor quality.

**Table 1:** Summary of baseline datasets including subject characteristics, basic hemodynamics, and procedural times

Subject characteristics				
Gender	[F:M]	10	:	10
Age	[years]	27	±	3
Mass	[kg]	72	±	15
Height	[m]	1.7	±	0.1
BMI	[kg/m^2^]	25	±	3
*Basic hemodynamics*				
HR	[bpm]	73	±	12
SBP	[mmHg]	116	±	13
DBP	[mmHg]	77	±	7
*Procedural times*				
Subj. charact./hemodyn.	[min]	10	±	1
Temp. measurements	[sec]	90^a^	±	15
IC	[min]	30	±	0
ICD^8°C^	[min]	30	±	0
ICD^15°C^	[min]	30	±	0
Experimental time/subject	[min]	100	±	0

Quantitative parameters are presented as mean ± SD

*BMI* body mass index; *HR* heart rate; *bpm* beats per minute; *mmHg* millimetres of mercury; *SBP* systolic blood pressure; *DBP* diastolic blood pressure; *IC* ice chips; *ICD*^*8°C*^ intraoral cooling device (ICD) set to 8 °C; *ICD*^*15°C*^ ICD set to 15 °C; *SD* standard deviation

^a^Each measurement

**Fig. 2:**
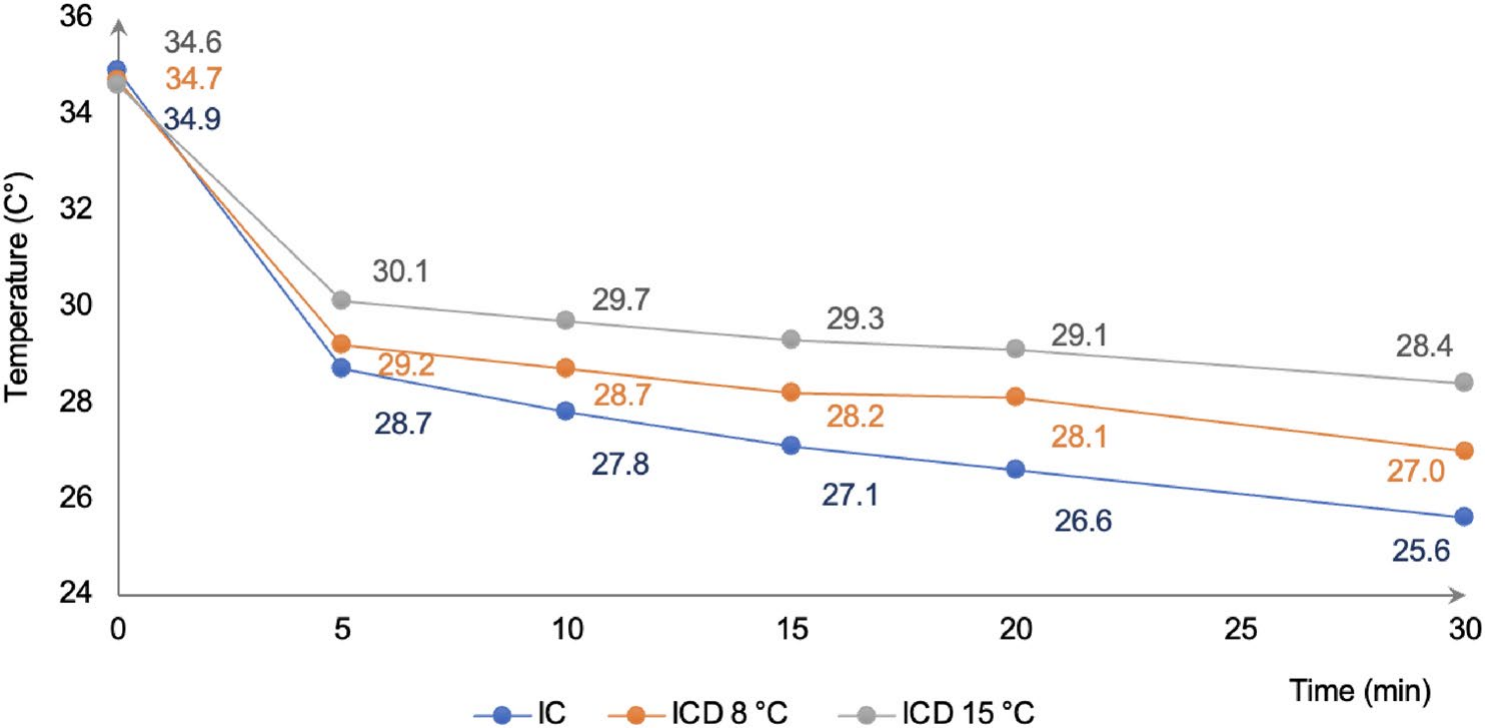
Intraoral mucosal temperatures at baseline and following 5, 10, 15, 20, and 30 min of cooling with ice chips (IC), and the intraoral cooling device (ICD) set to 8 and 15 °C, respectively

At baseline, when the surfaces of interest were grouped and assessed the mean intraoral temperatures were 34.9, 34.7; and 34.6 °C for ice chips (IC), intraoral cooling device set to 8 °C (ICD^8°C^), and intraoral cooling device set to 15 °C (ICD^15°C^), respectively. The observed differences of ≤ 0.3 °C between the three cooling methods did not reach a statistical significance (Fig. 2).

The greatest temperature reduction was reached following 5 min of cooling with all the tested methods, corresponding to 6.2 °C for IC, 5.5 °C for ICD^8°C^, and 4.5 °C for ICD^15°C^ as compared to baseline. The difference of 1.4 °C between IC and ICD^15°C^ after 5 min of cooling was statistically significant (*p* < 0.05). However, when the same comparison was made for IC vs. ICD^8°C^ and ICD^8°C^ vs. ICD^15°C^, the differences of 0.5 °C and 0.9 °C, respectively, did not reach statistical significance (Fig. 2).

The intraoral temperature further declined until cooling ceased. The lowest temperatures were therefore observed after 30 min of cooling, showing a temperature reduction of 9.3 °C for IC, 7.7 °C for ICD^8°C^, and 6.2 °C for the ICD^15°C^ as compared to baseline (Fig. 2). When data for the three modalities was compared at 30 min, a statistically significant difference was seen between IC and ICD^8°C^ (1.6 °C; *p* < 0.05), IC and ICD^15°C^ (3.1 °C; *p* < 0.001), and between ICD^8°C^ and ICD^15°C^ (1.5 °C; *p* < 0.05).

**Fig. 3:**
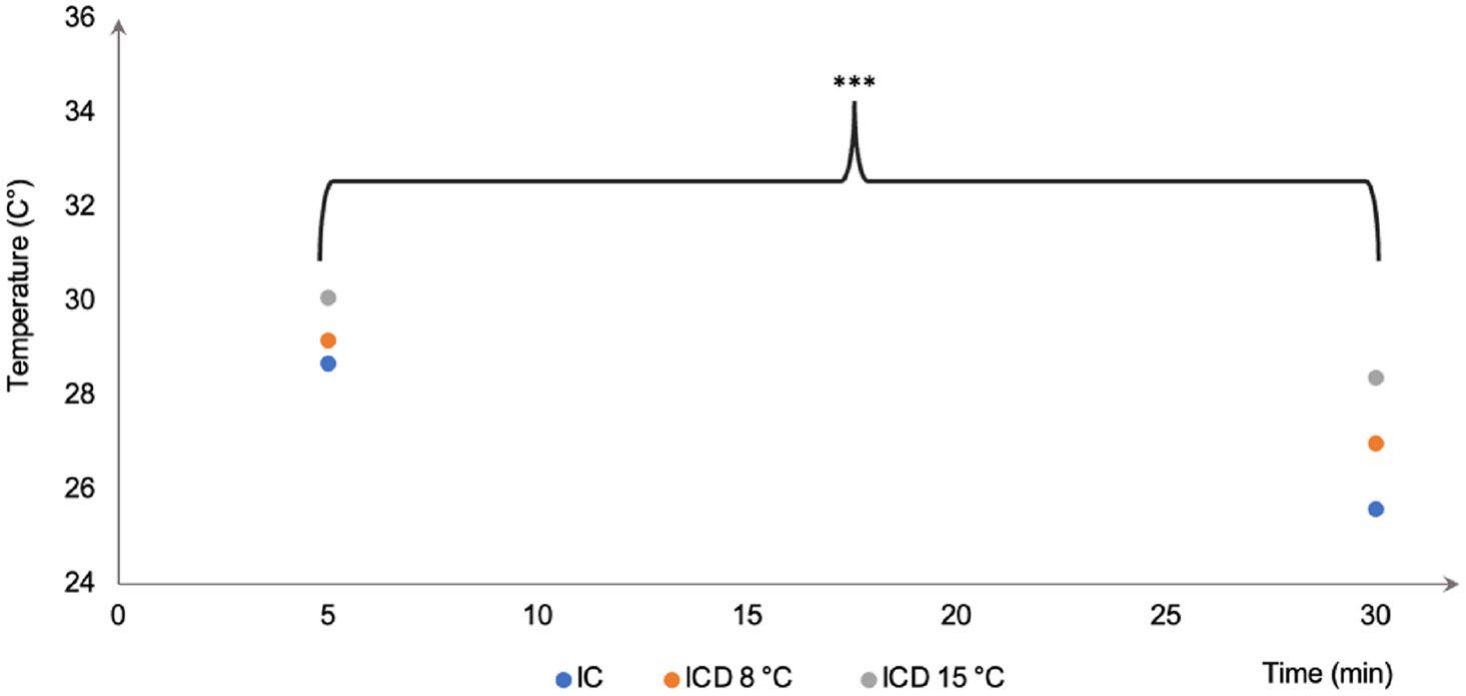
The difference in intraoral mucosal temperature reduction between 5 and 30 min of cooling within each cooling method, ice chips (IC), intraoral cooling device (ICD) set to 8 and 15 °C, respectively. ***p ≤ .001

**Fig. 4:**
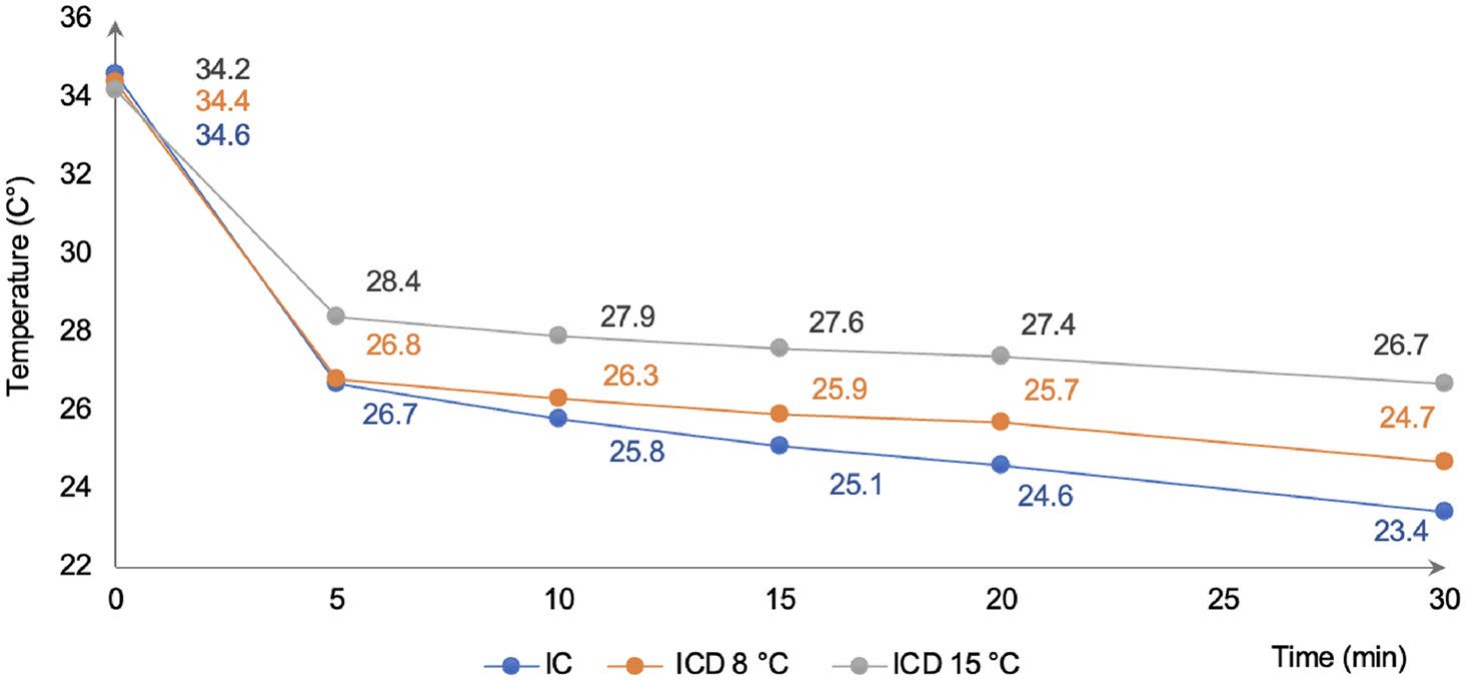
Intraoral temperatures for risk surfaces, i.e., right, and left buccal mucosae, upper and lower labial mucosae at baseline and following 5, 10, 15, 20, and 30 min of cooling with ice chips (IC), and the intraoral cooling device (ICD) set to 8 and 15 °C, respectively

The differences in temperature reduction between 5- and 30 min of cooling were 3.1 °C for IC, 2.2 °C for ICD^8°C^, and 1.7 °C for ICD^15°C^ (Fig. 3), reaching a statical significant difference for the three cooling methods (*p* < 0.001). Ultimately, the same analysis was repeated for the risk surfaces, i.e., the non-keratinized areas; right, and left buccal mucosae, upper and lower labial mucosae, displaying a similar pattern to that of when all surfaces were grouped and compared. However, at each of the follow up time points a more profound temperature reduction was observed, irrespective of cooling method used (Fig. 4).

## Discussion

Until recently, when a novel intraoral cooling device (ICD) was introduced, only modest resources had been devoted to further develop the modality of cooling in conjunction with chemotherapy. This is somewhat surprising since the superiority of OC for prevention of chemotherapy-induced oral mucositis (OM) has been demonstrated in several trials [16, 19, 26, 27]. Cooling with the ICD was equally effective but better tolerated when compared to ice chips (IC) in healthy volunteers [18]. Further, the ICD proved to enhance the efficacy of conventional IC in prevention of OM in patients with lymphoma, whereas efficacy results were comparable between the two cooling methods in the myeloma group. The collective tolerability data were in accordance with the previous study in healthy volunteers [19]. It was also shown that cooling with the ICD^15°C^ was better tolerated than the ICD^8°C^ but displayed inferior capacity in temperature reduction [28]. However, whether the discrepancy of approximately 2 °C is of clinical importance remains uncertain. Further, moderate temperature reduction was sufficient to reduce the early events which may precede clinically established OM. This could have clinical advantages in terms of tolerability [29].

Combined with existing evidence, it was reasonable to assume that cooling protocols in clinical settings should be further improved, especially with regards to when cooling should commence prior to chemotherapy infusion. Since it is believed that the lowest temperature achieved in the oral mucosa is the most protective, it was of importance to identify when along the course of cooling this is obtained, and if this is different depending on cooling modality and temperature. This was the rationale for conducting this study.

The greatest drop in intraoral temperature was seen after 5 min of cooling. This was true for all the tested cooling methods, i.e., IC, ICD^8°C^ and ICD^15°C^. The temperatures achieved in the oral mucosa after the initial drop is likely sufficient to prevent severe OM. This was confirmed in a randomized controlled trial where OC using IC started 5 min prior to the chemotherapy infusion and a low incidence of severe OM was reported [22].

Although, intraoral temperatures seemed to reach a plateau phase after the first 5 min of cooling, the temperatures continued to decrease during the following 25 min for all the tested methods, leading to a significant temperature decrease of approximately 2–3 °C. It is however justified to question if this additional decrease in temperature is of relevance for prevention of OM, and whether it outweighs the disadvantages of exposing patients to longer cooling sessions. A retrospective study including 134 patients scheduled for chemotherapy due to lymphoid malignancies, investigated the preventive effect of 40 min of OC with IC. Cooling commenced ten minutes prior to the chemotherapy infusion and showed significantly less grade III-IV OM, i.e., severe OM according to WHO-scale, as compared to the controls [30]. The efficacy of shorter cooling time was further assessed in a RCT where patients with hematological malignancies were randomized to either OC or saline mouthwash. OC was administered for a total of 30 min and initiated 5 min prior to the chemotherapy infusion and showed a significantly lower level of OM as compared to the controls [21]. The same pattern was seen in another study where OC was initiated 5 min prior high-dose chemotherapy and continued for a total of 40 min. OM was observed in 13/52 patients of whom 3/13 developed severe OM [31]. Thus, using shorter cooling periods prior to chemotherapy for OM prevention are comparable to when longer cooling sessions are used [19, 24, 27].

Considering this, 5 min of cooling prior to chemotherapy infusion appears to be enough to preserve the oral mucosa. This may be explained by the delicate histology of the intraoral mucosa [32, 33], in particular the superficial capillaries which seem to adapt promptly to intraoral temperature changes. As for the risk surfaces, i.e., the non-keratinized areas, a similar pattern was seen in terms of temperature reduction with all the modalities tested. However, with a more profound temperature reduction as compared to when all surfaces were grouped and analyzed. This should be considered an advantage, as chemotherapy-induced OM mainly manifests in these specific areas.

The main advantage of this study was that all images were analyzed by an observer blinded to the data sets. Thus, reducing the risk of bias. There are, however, some critical points of concern that should be acknowledged. First, this study was conducted on a limited number of healthy volunteers. Intraoral temperature reduction may be different in cancer patients subjected to cooling. A randomized controlled trial, including larger cohorts would be necessary to verify the results observed in this study. Second, the thermographic camera used in this study is not specifically designed for intraoral temperature recordings. Hence, the temperature recordings may not reflect actual intraoral temperatures. Nonetheless, as the same method was used throughout the study, the potential limiting factor may have affected all cooling methods equally.

## Conclusions

The greatest drop in intraoral temperature is seen following 5 min of cooling. Additional cooling time further decreases the intraoral temperatures, but whether this is of clinical significance to prevent OM remains to be elucidated.

## Data Availability

The datasets generated and analyzed during the current study are available from the corresponding author on reasonable request.
